# PARma: identification of microRNA target sites in AGO-PAR-CLIP data

**DOI:** 10.1186/gb-2013-14-7-r79

**Published:** 2013-07-29

**Authors:** Florian Erhard, Lars Dölken, Lukasz Jaskiewicz, Ralf Zimmer

**Affiliations:** 1 Institut für Informatik, Ludwig-Maximilians-Universität München, Amalienstrasse 17, 80333 Munich, Germany; 2 Department of Medicine, University of Cambridge, Box 157, Level 5, Addenbrooke's Hospital, Hills Road, CB2 0QQ Cambridge, UK; 3 Biozentrum, University of Basel and Swiss Institute of Bioinformatics, Klingelbergstr. 50/70, CH-4056, Basel, Switzerland

**Keywords:** PAR-CLIP, microRNA, generative model, EM algorithm

## Abstract

PARma is a complete data analysis software for AGO-PAR-CLIP experiments to identify target sites of microRNAs as well as the microRNA binding to these sites. It integrates specific characteristics of the experiments into a generative model. The model and a novel pattern discovery tool are iteratively applied to data to estimate seed activity probabilities, cluster confidence scores and to assign the most probable microRNA. Based on differential PAR-CLIP analysis and comparison to RIP-Chip data, we show that PARma is more accurate than existing approaches. PARma is available from http://www.bio.ifi.lmu.de/PARma

## Background

MicroRNAs (miRNAs) are important post-transcriptional regulators in all known multicellular organisms. These 20- to 24-nucleotide-long RNA molecules play roles in development, tumorigenesis and viral infection [1]. Generally, they bind to 3' UTRs of their target transcripts inhibiting translation or inducing degradation of the target mRNA [2]. Neither the exact mode of binding nor the mechanisms of downregulation are completely understood and these are being heavily debated [3-7]. It is believed that miRNAs recognize their target sites using only a few bases at their 5' end called the seed [8] and that other factors, such as additional base pairing at the 3' end [2], target site accessibility [9], target site location and AU content around the target site, contribute to recognition [10]. These factors, as well as the evolutionary conservation of target sites (for conserved miRNAs), have been used to predict target sites of miRNAs [11,12]. However, all known prediction methods are hampered by a huge number of false positives and false negatives [13]. Recently, several high-throughput assays have been developed, which allow accurate identification of miRNA targets (reviewed in Thomson *et al. *[14]).

Immunoprecipitation (IP) of the Argonaute (AGO) protein, the major component of the RNA-induced silencing complex (RISC), is used to identify the miRNA-mediated recruitment of hundreds of different transcripts to the RISC. Target mRNAs of miRNAs co-precipitate with AGO and can thus be identified using either microarrays (RNA binding protein immunoprecipitation followed by chip analysis (RIP-Chip)) or next-generation sequencing (RNA binding protein immunoprecipitation followed by sequencing analysis (RIP-seq)) [15-20]. However, these RIP experiments only give information about target genes or transcripts and do not give the precise location of target sites nor the actual miRNA targeting these sites. As a result, novel techniques including high-throughput sequencing of RNA isolated by cross-linking immunoprecipitation (HITS-CLIP), individual nucleotide resolution cross-linking and immunoprecipitation (iCLIP) and photoactivatable ribonucleoside-enhanced cross-linking and immunoprecipitation (PAR-CLIP) have been developed. Before the IP, RNA is cross-linked to proteins using UV light, and the precise location of the target site is determined by deep sequencing of cross-linked RNA after digestion of non-cross-linked RNA [21-23]. The actual miRNA binding at these sites still has to be determined.

Both techniques, RIP and CLIP, need specialized bioinformatic analysis methods. RIP is very similar to standard gene expression experiments and, thus, advanced analysis methods are readily available. In addition to these standard approaches, in a recent paper, we described additional algorithms that need to be employed to consider and cope with the characteristic features of RIP data [24]. In contrast, CLIP data are more complex: first, short sequencing reads must be aligned to the genome or transcriptome and then clustered [21-23]. True target sites have to be identified in the clusters and the specific miRNA targeting each site has to be determined. Depending on the exact experimental protocol, true target sites may look quite distinctive: for HITS-CLIP, narrow peaks in the read coverage are expected [21], while iCLIP clusters show specific read start positions [22] and PAR-CLIP clusters are characterized by T to C conversions [23]. Here, we focus on PAR-CLIP, a technique that has been used by several groups to identify miRNA target sites [23,25-27].

In their original PAR-Clip paper, Hafner *et al. *[23] used several manually chosen parameters to define target sites (for example, at least two distinct conversion positions per cluster and at least five sequencing reads). They recognized that the region downstream of the main conversion site is enriched for sequences complementary to the seeds of the top expressed miRNAs.

PARalyzer is a software package specifically designed to define RNA binding sites from PAR-CLIP data. Reads are first clustered and filtered using similar parameters as those used by Hafner *et al. *[23]. Then, conversion and non-conversion distributions are computed by counting the respective events and employing kernel density estimation along each cluster. All positions with a higher conversion than non-conversion density are considered target sites and surrounding sequences are submitted to a standard motif discovery tool that uses linear regression to determine miRNA seed sites enriched among clusters with many conversion events [28].

There are several open problems in PAR-CLIP data analysis: first, it is unclear which miRNAs should be taken as a starting point for searching seed sites in PAR-CLIP clusters. In all published studies, the top *N *miRNAs according to miRNA read counts in the PAR-CLIP experiment or an additional experiment are taken. However, read counts provide a potentially strongly biased estimate of miRNA expression levels [29,30]. In addition, it is unclear how many miRNAs should be used. Finally, it may not be sufficient to consider only known miRNAs: first, there are indications that there are still many unknown miRNAs [31] and second, not only miRNAs (as defined by their maturation pathway) may be associated with AGO and used for target recognition, since there may be other pathways that lead to the incorporation of small RNAs into RISC [32-36].

Second, the specific information given by a PAR-CLIP experiment is only partially exploited: in the PAR-CLIP protocol, RNase T1 is used to digest RNA, which cleaves specifically after guanine [37]. This information could be used to exclude seed sites spanning read start or end positions under the assumption that these sites are protected from digestion by the miRNA. Also, it is known that positions in the mRNA bound to the miRNA cannot be efficiently cross-linked and, thus, seed sites spanning a cross-linking site could also be excluded [23]. Currently, there is no method available that directly uses the information from RNase cleavage sites or conversion sites for the discovery of motifs or the assignment of seed sites. Third, no available scoring system has been demonstrated to identify clusters or assigned miRNAs reliably.

Here, we present a method to address these aspects: PAR-CLIP miRNA assignment (PARma) seeks explanations for the presence of each identified PAR-CLIP cluster. Here, an explanation is a *k*-nucleotide-long sequence (a k-mer) within a cluster that corresponds to the seed of the miRNA binding this site. PARma explains each PAR-CLIP cluster by a k-mer that (a) explains multiple clusters with high probability and (b) matches a generative model for the experimental data (that is, the data observed in the experiment are likely to be generated by amiRNA binding at the determined position). The determined k-mer can identify miRNA families that are characterized by a seed matching the k-mer. The model is able to score each k-mer in a cluster according to the observed conversions and RNase cleavage sites. Parameters as well as k-mer activity probabilities are estimated in an iterative manner. The model assigns the most probable seed to each PAR-CLIP cluster, scores each cluster according to the confidence to correspond to a true miRNA target site and also scores the confidence of the assignment of the correct seed.

Differential PAR-CLIP data was used to evaluate our methods: we used pairs of PAR-CLIP datasets with miRNAs that are known to be present in the first dataset but not in the second. When these pairs are analyzed, the target sites (PAR-CLIP clusters) of these miRNAs should only be present in the first dataset. We used our own PAR-CLIP datasets of the two B-cell lines DG75 and BCBL1, of which only the latter is infected with Kaposi's sarcoma-associated herpesvirus (KSHV), a herpesvirus encoding 25 mature miRNAs. In this data, we expect the viral miRNAs, and hence their targets, only to be present in the infected cell line. We also repeated our evaluations using a published dataset of positive and negative cell lines for the Epstein-Barr virus (EBV), which encodes 44 mature miRNAs [25].

## Results

### PARma overview

**Figure 1:**
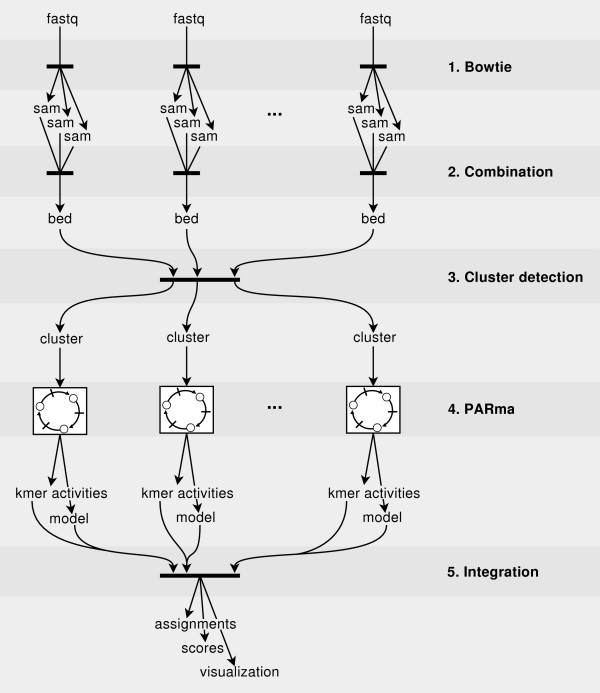
**PAR-CLIP data analysis pipeline**. The PARma workflow starts with the raw data from PAR-CLIP experiments (replicates or different conditions), that is, several fastq files containing sequencing reads. First, we utilize Bowtie [46] to align these reads to multiple reference sequences such as the human genome and transcriptome or viral genomes, which results in several sam files, one for each fastq file and reference sequence. Second, for each read from each experiment we identify all optimal alignments in terms of mismatches, considering T to C conversions as matches, and map transcriptomic reads that span splice junctions to the genome. Third, possible target sites of miRNAs are identified by clustering reads from all datasets simultaneously. The clusters including additional annotations such as the number of conversions and cleavages per position are written to separate files for each experiment. The cluster detection module implements a splitting procedure to identify target sites with overlapping reads and is able to handle target sites that span splice junctions. Fourth, for each dataset, the core PARma component estimates a generative model for the data and k-mer activity probabilities using kmerExplain in an iterative manner (see also Figure 3). Fifth, the models and the activity probabilities are used to score clusters and to assign the most probable miRNA. Target sites with various annotations such as gene ids are written to tabular files that can be further analyzed and visualized.

**Figure 2:**
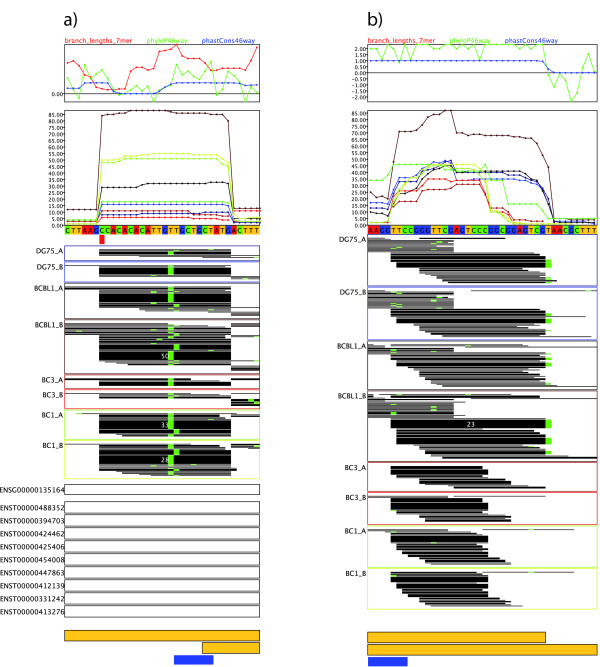
**PAR-CLIP data viewer**. From top to bottom, both panels show conservation scores (branch lengths of seven-mers as described by Friedman *et al. *[11] and the widely used phyloP [47] and phastCons [48] scores, all computed for the 46-way vertebrate multiple alignment obtained from the UCSC genome browser [49]), the read coverage in each experiment and the genomic sequence of the cluster. Below the sequence, SNP positions according to the 1000 genomes project are indicated in red (here there is only one in (a)) and the actual sequencing reads are shown as black bars for each of the experiments. Mismatches are color-coded as in the genomic sequence above (in both clusters, there are only T to C conversions). Different sequences that have been mapped to a cluster can be distinguished by distinct start or end positions of the corresponding bars or distinct mismatches. The height of each bar is proportional to the corresponding read count. For clarity, if a sequence is observed more than 15 times in an experiment, the corresponding bar is not heightened further and the read count is indicated in white. Ensembl genes and transcripts are shown below the reads (here these are present only in (a)), together with PAR-CLIP clusters in yellow and seed site assignments in blue. **(a) **An experimentally validated target site of hsa-miR-l5 in the 3' UTR of DMTF1. This illustrates the characteristic features of many valid target sites (see main text). Interestingly, there is also a known SNP (red box) in proximity to the seed site. **(b) **An intergenic (that is, there are no Ensembl genes or transcripts) cluster that does not have these characteristics. Additionally, it does not contain a miRNA seed site nor any overrepresented seven-mer according to PARma. The validated cluster has Cscore and MAscore > 0.9, whereas for the intergenic cluster, both scores are 0.

We developed a complete workflow for the analysis of PAR-CLIP data (see Figure 1). The main steps are: (a) mapping of the sequencing reads to reference sequences, (b) detection of read clusters corresponding to target sites, (c) estimating a model that represents characteristic features of the PAR-CLIP data and miRNA (seed) activities and (d) the final assignment of miRNAs to target sites and scoring using the derived model. Furthermore, we developed a tailored, web-based visualization for PAR-CLIP data, which helped us during the development of PARma and can be used to investigate manually specific target sites (see Figure 2).

**Figure 3:**
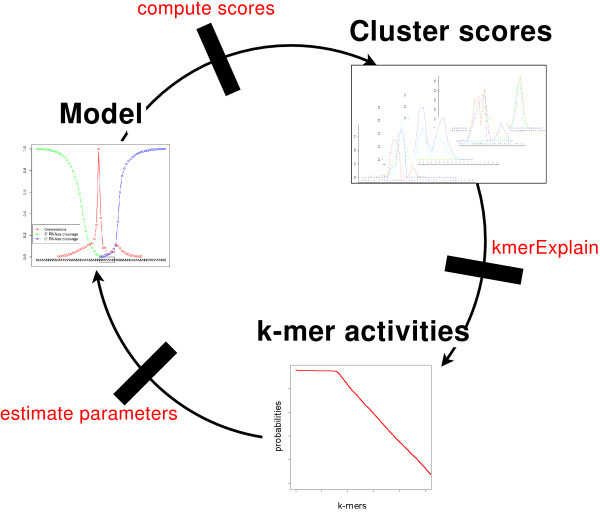
**How PARma works**. PARma is an iterative algorithm, which repeatedly executes three steps: based on a current model of the PAR-CLIP characteristics (left; see also Figure 6), scores are computed for each position in each cluster, which express the likelihood that the cluster is explained by the activity of the k-mer at this position (top right; see also Figure 7). These scores are fed into kmerExplain as prior probabilities, which then estimates k-mer activity probabilities using an EM algorithm (bottom). These k-mer activities in conjunction with data from the PAR-CLIP experiment (T to C conversions and RNase cleavage sites) are used to estimate the parameters of the PAR-CLIP model. We start this procedure by running kmerExplain on uniform scores and end it as soon as the model converges. EM, expectation maximization.

The central idea of PARma is that miRNAs binding to a target site will generate specific data in a PAR-CLIP experiment (conversion positions and RNAse T1 cleavage sites, see Figure 2a). Thus, given experimental data and a model representing these features, it is possible to infer the binding site with the highest likelihood of generating these data. Additionally, given the experimental data and the correct binding sites, it is straightforward to infer the model parameters. Thus, we are facing a chicken-or-egg dilemma: if we knew the binding sites we could infer the model, and if we knew the model, we could infer the binding sites. In PARma, this is resolved using an iterative procedure (see Figure 3). We start by computing statistically overrepresented k-mers in clusters and take these as initial estimates for the correct binding sites. Then, we infer model parameters and iteratively refine all estimates until convergence. During these iterations, seed activity probabilities are estimated, corresponding to the likelihood-weighted number of target sites. Importantly, it is possible - but not necessary - to specify an *a priori *set of allowed miRNAs. This is a highly desirable feature since in general it is not known which miRNAs are active in an experiment, and the read count of the miRNAs themselves in the PAR-CLIP experiment or an external sequencing experiment is only a weak proxy for their activity, as shown below.

In PARma's final output, for each cluster the most probable seed is assigned, together with a cluster score (Cscore) and a miRNA assignment score (MAscore). The Cscore indicates how well the observed data (conversions and RNase cleavage sites) fit the model without considering the k-mer probability and therefore it indicates whether an observed cluster is indeed a true miRNA target site. The MAscore corresponds to the confidence of the assignment, that is, whether there are other active k-mers in the cluster that also match the observed data well.

### Cluster detection

After read mapping (see Methods), the first main step of PAR-CLIP data analysis is to identify clusters of reads corresponding to target sites. We use a procedure that is similar to one that has been used previously with a few but important modifications. First, PARma is able to search for clusters using multiple datasets simultaneously. This not only increases sensitivity, but also provides a straightforward way for a differential analysis of target sites, since it is not necessary to identify corresponding clusters from two or more experiments after individual processing. During cluster identification, clusters are determined for all datasets simultaneously, and each cluster is quantified for each dataset.

**Figure 4:**
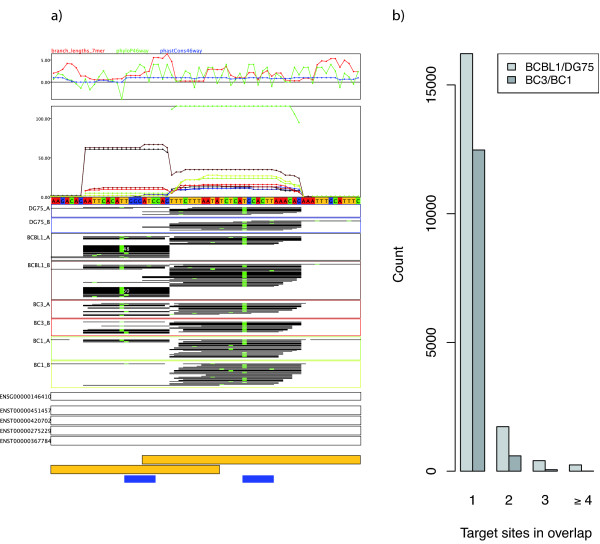
**Overlapping PAR-CLIP clusters**. **(a) **Two target sites that would fall into the same cluster by the definition of Hafner *et al. *[23], only because in the two DG75 replicates as well as in the second BCBL1 replicate a few random reads from the right target site overlap the left target site. Our cluster definition splits all reads into two overlapping clusters (see the yellow boxes on the bottom). PARma rates both clusters with high Cscores (>0.6 and >0.9 for the left and right clusters, respectively) and assigns the KSHV miRNA kshv-miR-Kl2-7 to the left and the human miRNA hsa-miR-5l9 to the right cluster with MAscores >0.9 in both cases. There is additional evidence that both assignments are correct, since the left cluster has reads only in KSHV positive cell lines (BCBL1, BC1 and BC3) whereas the right cluster contains reads in all experiments. **(b) **There are hundreds of such cases in both experiments. KSHV: Kaposi's sarcoma-associated herpesvirus.

Second, the original definition of PAR-CLIP clusters (that is, target sites) by Hafner *et al. *[23] involved a single linkage clustering of overlapping reads. However, we observed several cases where such a procedure tends to link multiple target sites into a single cluster due to few spurious reads that connect two obviously distinct clusters (see Figure 4a for an example). Such cases are relatively frequent (see Figure 4b) and may be of special interest: for instance, there are known cases where viral miRNAs bind to sites in a neighborhood close to target sites of human miRNAs [38]. Missing individual clusters due to overlapping reads would be detrimental to such an analysis. Thus, we devised a cluster-splitting procedure, which is able to detect such cases effectively.

And third, we align PAR-CLIP reads to the transcriptome as well as the genome. Transcriptomic reads are then mapped to genomic coordinates and may therefore produce spliced reads. These are properly respected during cluster detection, that is, PARma is able to detect target sites spanning exon-exon junctions. In previous studies using AGO-PAR-CLIP data [23,25-27], this was not considered, and several highly interesting target sites were probably missed. Indeed, in the datasets we analyzed, 22.4% of all clusters in the coding region of transcripts span splice junctions (about 6% of all clusters).

### Generative model

The novel feature in PAR-CLIP (in comparison to other CLIP protocols) is the use of the uridine analogue 4-thiouridine, which is not read as U but as C during cDNA synthesis following its cross-linking to proteins [23]. Thus, T to C mismatches of aligned sequencing reads are characteristic of cross-linked sites and, therefore, for contacts of the examined protein with RNA. Since RNase T1 is used in the PAR-CLIP protocol, which cleaves specifically downstream of guanine, it is important where sequencing reads start and end. In most cases, the RNase products are shorter than the sequencing reads (36 for Gottwein's data [25] and 50 for our data). Therefore, in these cases the complete RNA fragments are known.

Visual inspection of these features for known target sites of miRNAs using our PAR-CLIP data browser (see Figure 2) showed several characteristics of these targets sites that go beyond the characteristics of individual PAR-CLIP sequencing reads (see Figure 2a): in most cases, there is a main cross-linking site and ≥60% of all conversions in the cluster belong to this site, a fact that has been recognized before [23]. In addition, this main cross-linking site tends to lie in the center of most sequencing reads and T sites upstream tend to be cross-linked more often than T sites downstream of the main site. Another well-established feature is the position of seed sites preferentially downstream of the main cross-linking site. Finally, in addition to these main cross-linking sites, there are main RNase cleavage sites with specific locations: one is approximately ten to twenty nucleotides upstream of the seed site, the other usually immediately downstream of the seed site. While the upstream cleavage site often skips several G sites, the downstream site is, in most cases, immediately after the next G. To represent these features formally, we developed three independent probabilistic models: the conversion model and the upstream and downstream cleavage models. Given the position of a seed site and the positions of uridines or guanosines, respectively, each model is able to predict where and how many conversions or cleavages, respectively, would be generated by a PAR-CLIP experiment. By comparing the predicted data to the measured data, a likelihood for each possible seed position within a cluster can be computed. Specifically, the conversion model generates many conversions directly upstream of a seed position (given there is a uridine), and almost no conversions within the seed. Thus, such a position would receive a high score only if this is indeed observed in the experiment.

Model parameters (for example, how many conversions are expected for each uridine within a cluster) are directly learned from the data per experiment using robust parameter estimation techniques. Doing this for each dataset individually is important, since experimental conditions may be slightly different between experiments, potentially leading to slightly different data per cluster.

### KmerExplain

KmerExplain optimizes a probabilistic modelunder the assumption that each target site is targeted by a single miRNA family, that is, each cluster must be explained by a single k-mer (that is, miRNA seed). There are two conditions for the explaining k-mer implicated by the model: first, its position in the cluster has to match the generative PAR-CLIP model, that is, the given data (conversions and cleavages) are likely to be generated by a seed matching to this position. And, second, the k-mer has to be likely to be active, that is, there are many instances where this k-mer explains a cluster. The model is fitted with an expectation maximization (EM) algorithm.

### Seed activities

We applied PARma to a previously published PAR-CLIP dataset consisting of two replicates for each of the B-cell lines BC3 and BC1, as well as to our own PAR-CLIP data consisting of two replicates for each of the B-cell lines DG75 and BCBL1. First, we analyzed the correlation of miRNA expression as measured by its PAR-CLIP read count and its activity as measured by the number of assigned target sites.

**Figure 5:**
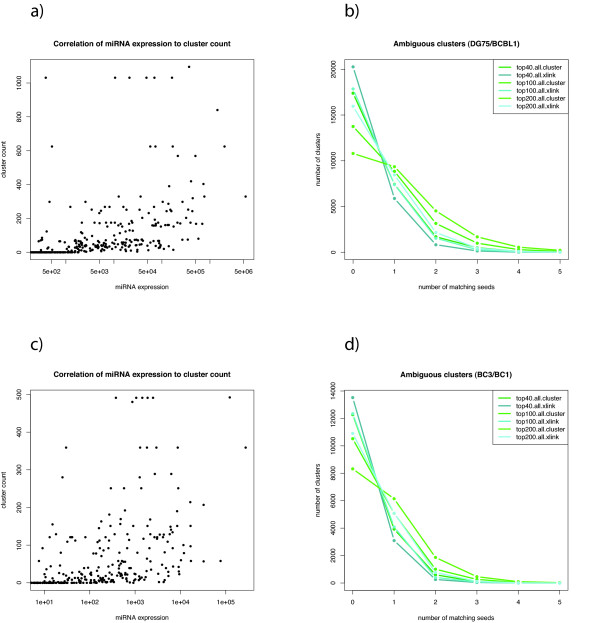
**Correlation of miRNA expression to the number of assigned clusters**. Here, miRNAs have been assigned to a cluster when they are among the top 200 expressed miRNAs and match the first seed site downstream of the main cross-linking site. Neither the BCBL1 PAR-CLIP data **(a) **nor in the BC3 PAR-CLIP data **(c) **show strong correlation. **(b) **and **(d) **illustrate how many seven-mer seeds match to clusters when the top 40,100 and 200 miRNAs are considered and when seeds are searched in the whole cluster (*all*) and only downstream of the main cross-linking site (*xlink*). Even the strictest assignment (*top 40 xlink*) leads to a considerable number of approximately 1,000 ambiguous clusters in both datasets and at the same time to about 80% unassigned clusters. The fraction of unassigned clusters drops below 50% when the top 200 miRNA seeds are searched in the whole cluster but with the cost of having thousands of ambiguous assignments.

Even if it is true that the top 100 expressed miRNAs explain >50% of the clusters using a six-mer seed, the overall correlation between miRNA expression and the number of corresponding target sites is poor (see Figure 5). This is a general observation and does not depend on how miRNAs have been assigned to clusters (a variety of options have been explored: all seed sites in the cluster, a random seed site in the cluster, the first or a random seed downstream of the main cross-linking site, using the top 40, 100 or 200 miRNAs and six-mer or seven-mer seeds). The poor correlation may be a consequence of sequencing artifacts, which are known to bias expression estimates of miRNAs significantly [29,30].

In addition, we and others proposed that not only miRNAs may enter the RISC pathway, but there may be other maturation pathways producing small RNA molecules, which could act analogously to miRNAs in RISC [31-36,39,40]. Furthermore, even if only the seven-mer seeds of the top 40 miRNAs are used and seed sites are only considered when downstream of the main cross-linking site, there are hundreds of clusters where two or more seeds match. Necessarily, this issue becomes more severe, if more than 40 miRNAs or all seed sites within a cluster are used (see Figures 5b and 5d).

Taken together, these facts suggest that the paradigm of taking the top *N *expressed miRNAs as candidate regulators for PAR-CLIP clusters should be abandoned. Therefore, we designed PARma to identify k-mers among all possible 4*^k ^*k-mers that can explain multiple clusters with high probability. Furthermore, as well as explaining multiple clusters, their positions must be in agreement with the model derived from the data for all clusters.

### Inferred models

**Figure 6:**
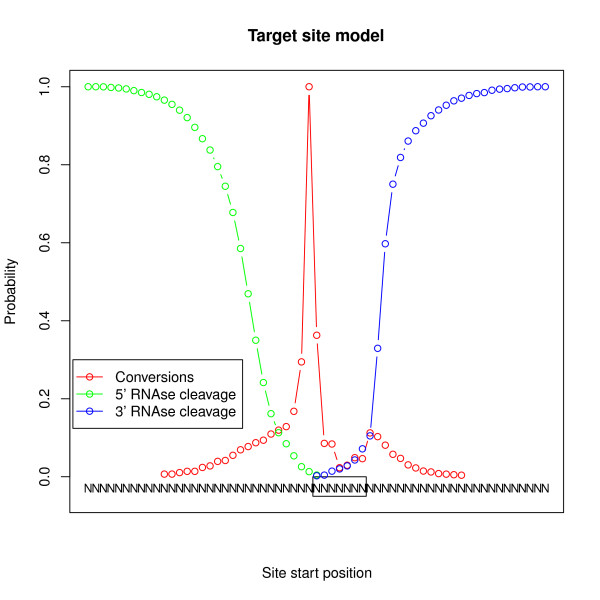
**PARma model for replicate A of the DG75 experiment**. The conversion model in red represents the conditional propensity that a base becomes cross-linked given there is a uridine at the corresponding position. Note that the propensity is only known up to a constant factor and arbitrarily scaled to a mode of 1. The blue and green lines illustrate the 3' and 5' cleavage models, respectively. These correspond to the conditional probabilities that the RNase Tl cleavage site is at a certain position or closer to the seed site given that there is a guanine. The model shows that the observations made for a few visually inspected validated target sites are also true globally for many clusters.

Next, we analyzed the generative model estimated by PARma. In Figure 6, the model for replicate A of DG75 is illustrated. It indeed reflects the above mentioned observations: the conversion model indicates the expected ratios of conversions around the seed site for all positions where a T is located. For instance, if there is a T immediately upstream of the seed site and a T immediately downstream, the expected ratio of conversions is about 10:1. Furthermore, the first position in the seed site also seems to become cross-linked with relatively high frequency (for an example, see Figure 2a).

The models from Figure 6 are in agreement with what is known of miRNA target recognition [2]: a canonical miRNA binding site consists of a seed site complementary to the miRNA seed (bases two to seven or two to eight), often base one is the opposite of an A and often there is additional base pairing of the miRNA 3' end after a small loop. Thus, the seed site itself may be protected from cross-linking by the seed, bases immediately upstream of the seed are accessible and further upstream bases may also be protected by the miRNA 3' end to some extent.

Furthermore, the model also agrees with structural features of AGO[41]: miRNA bases two to six are solvent exposed and there is a distinct kink separating bases six and seven, which may be resolved by conformation changes of AGO [41]. These conformation changes may be a reason for the relatively high cross-linking probability of the first position of the seed site. Another explanation is that PARma may find several instances of 7mer-m8 seed sites (pairing of bases two to eight) as well as 7mer-A1 seed sites (pairing of bases two to seven plus an A opposite base one). The first base of the identified k-mer may therefore be opposite base seven or eight of the miRNA, and, therefore, may or may not be accessible for cross-linking.

**Figure 7:**
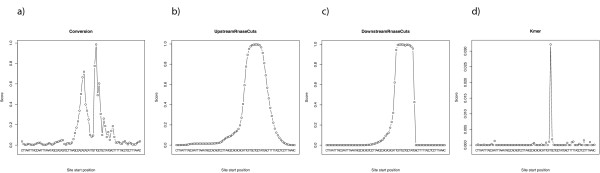
**Model scores for the cluster in Figure 2a**. Each graph shows how well one of the submodels of PARma matches when aligned to the seven-mer that starts at the corresponding position. For instance in **(a)**, the maximal value belongs to the seven-mer TGCTGCT and indicates that all observed and unobserved T to C conversions match very well, when TGCTGCT is the miRNA seed site. A miRNA targeting the seed site CACATTG (corresponding to the secondary peak upstream of TGCTGCT) is also likely to lead to the observed conversion. The cleavage scores in **(b) **and **(c) **indicate how likely the observed RNase Tl cleavages are, given the seed site is at the corresponding position. Both submodels would allow seed sites to start within a small window of about 10 bases and indicate that the secondary peak from (a) is unlikely to correspond to the true miRNA seed site. However, they agree with the primary peak of the conversion scores. Finally, the k-mer activity scores in **(d) **indicate how many other PAR-CLIP clusters are likely to be explained by the corresponding k-mer and they also point to the seven-mer TGCTGCT. This is indeed the seven-mer-m8 seed site for miR-l5a, and it has been experimentally validated that it targets this cluster [42].

As described above, all three submodels can be used to compute a score for each possible seed site position within a cluster. The conversion score (see Figure 7a for the cluster in Figure 2a) indicates that likely positions for a seed site are either immediately upstream or downstream of the main cross-linking site. The downstream position is obvious; the upstream position, however, is also probable, since further upstream there is no T that could get cross-linked. Figures 7b and 7c illustrate that the seed position is restricted to a small part of the cluster due to the clear 5' and 3' RNase cleavage sites. In addition, based on the estimate from kmerExplain, the k-mer TGCTGCT (see Figure 7d) is highly active and indeed corresponds to the 7mer-m8 seed site of the miR-15/16 family, which is highly expressed in B-cells. Hence, PARma is able to predict the corresponding position with high confidence, and, indeed, it is an experimentally confirmed target site of miR-15a [42].

Although the PAR-CLIP protocol is rather stringent and thus provides reasonably pure AGO complexes, other RNA-protein interactions of co-purified proteins or abundant cellular proteins may be responsible for cross-linked and protein-protected RNA fragments, giving rise to non-AGO PAR-CLIP clusters. The model we developed is also used to compute a cluster score (Cscore), which indicates the likelihood that a given cluster actually represents a miRNA binding site, that is, how well the observed data (conversions and RNase cleavage sites) fit the model without considering the k-mer probability. The miRNA assignment score (MAscore) indicates whether there are other overrepresented k-mers in the cluster that also match the observed data well. The experimentally confirmed target site in Figure 2a has Cscore and MAscore of 0.9608 and 0.9777, respectively, whereas the cluster in Figure 2b has a Cscore of 0, indicating that there is no position where conversions and RNase cleavage sites agree.

### Evaluation using differential PAR-CLIP

We evaluated PARma against PARalyzer and the standard approaches for assigning seeds for the top *N *miRNAs (for *N *= 40, 100 and 200) when they occur somewhere in a cluster (*cluster*) or downstream of the main cross-linking site (*xlink*) and either assigning every seed (*all*) or a random/the first seed (for *cluster *and *xlink*, respectively), when there are multiple seeds present. For the evaluation, we exploited a unique feature of the datasets we used: in our own data, only the cell line BCBL1 and not DG75 is infected by KSHV, which encodes 25 mature miRNAs, some of which are highly expressed in BCBL1 [20]. Thus, PAR-CLIP clusters that are assigned to one of the KSHV miRNAs should not be present in DG75 and we can use the number of KSHV-assigned PAR-CLIP clusters in DG75 as a measure of assignment accuracy. Although both cell lines, BC3 and BC1, in the PAR-CLIP data from [25] are infected by KSHV, only BC1 is co-infected by EBV, which encodes 44 mature miRNAs. Hence, PAR-CLIP clusters that are assigned to one of the EBV miRNAs should not be present in BC1.

**Figure 8:**
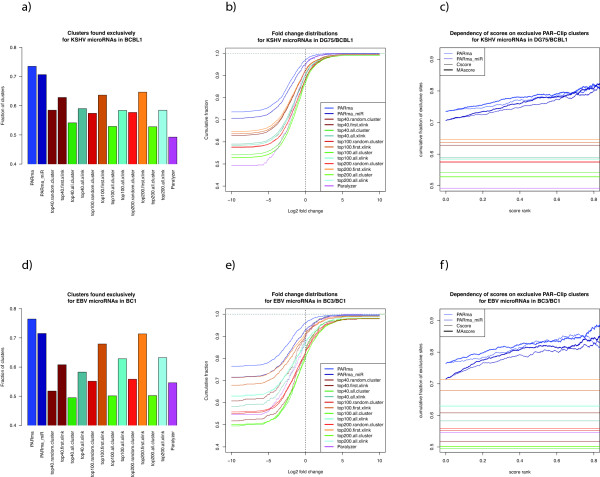
**Evaluation using differential PAR-CLIP**. KSHV miRNA target sites should only have reads in KSHV-infected cell lines **(a-c)**, and EBV miRNA target sites should be exclusive to EBV infected cell lines **(d-f)**. PARma-assigned KSHV miRNA target sites have a higher fraction of exclusive sites than any other method (a, d) (see main text for a description of the other methods) and when PARma was run without being constrained to known miRNA seeds, it yielded a higher fraction of exclusive sites than PARma using seeds as priors. (b) and (e) show the log fold changes (control/infected) of PAR-CLIP read counts for clusters assigned to KSHV and EBV miRNAs, respectively. The log fold change of exclusive clusters (that is, clusters that have no reads in one of the experiments) has been set to -10 or 10. PARma not only has the largest fraction of exclusive clusters in both datasets (compare the left ends of (b) and (e) to (a) and (d), respectively) but it also has the smallest fraction of KSHV or EBV clusters that have more reads in the KSHV or EBV negative cell line. The dependency of scores on the accuracy is shown in (c) and (f). In both datasets and for both scores, accuracy increases as low scoring clusters are removed. As a reference, the accuracies of the other assignment methods are indicated with the same colors as in (b) and (e). EBV: Epstein-Barr virus; KSHV: Kaposi's sarcoma-associated herpesvirus.

With respect to exclusive sites, PARma is more accurate than all other methods, including PARalyzer, independent of the dataset used for evaluation (see Figures 8a and 8d). More than 70% of all clusters, where PARma assigned a KSHV or EBV miRNA, only have reads in BCBL1 or BC1, respectively. This number drops to about 50%, when any seed match of a KSHV miRNA in a cluster is taken as evidence for a KSHV target site (*all.cluster*) or PARalyzer is used. When a seed match immediately downstream of the main cross-linking site is used (*first.xlink*), the accuracy is almost as high as for PARma, but is heavily dependent on both dataset and the number of miRNAs used. Additionally, PARma's accuracy is significantly higher when it is run starting with all 16,384 seven-mers (PARma) instead of miRNA seven-mer seeds only (PARma_miR). This suggests that in several cases there are seeds of KSHV/EBV miRNAs in a non-exclusive cluster but there are also other overrepresented seven-mers that explain the conversions and RNase cleavage sites better.

We noticed that often random reads are scattered across expressed transcripts in all experiments. Thus, a true KSHV miRNA target site may have random reads in the KSHV negative cell line (DG75) and, therefore, may not be exclusively present in BCBL1. Therefore, we considered the number of PAR-CLIP reads in each KSHV or EBV miRNA assigned cluster and plotted their log fold change of DG75/BCBL1 or BC3/BC1, respectively (see Figures 8b and 8e). Independent of the fold change cutoff, PARma consistently identifies more KSHV or EBV miRNA clusters that have less reads in DG75 than in BCBL1 or in BC3 than in BC1, respectively. Specifically, less than 5% of KSHV clusters have more reads in DG75 than in BCBL1 for PARma assignments, which drops to below 90% for the other assignments.

In order to evaluate the computed Cscores and MAscores (see Methods section), we sorted clusters according to Cscore or MAscore and computed the fraction of BCBL1 and BC1 exclusive sites for KSHV and EBV miRNA assigned clusters, respectively. For both datasets the accuracy increases as the low scoring clusters or clusters with multiple possible miRNAs are removed, and accuracies of 80% or more were achieved (see Figures 8c and 8f).

### Validation against RIP-Chip data

To further validate target sites and target site assignments that are only found by PARma, and to invalidate target sites that were not detected by PARma but by other methods, we considered RIP-Chip data that we measured for the cell lines DG75 and BCBL1 [20]. In the RIP-Chip experiments, the amount of an RNA co-immunoprecipitated using an anti-AGO2 antibody was compared to RNA from a control IP using microarrays. Thus, this quantitatively measures the recruitment of an mRNA to Ago2-complexes and is an alternative technique to PAR-CLIP to determine miRNA targets. Using the right data analysis methods [24], the differential enrichment of mRNAs with RISC can be computed between BCBL1 and DG75, which indicates whether an mRNA is more strongly associated with RISC in BCBL1 than in DG75. On average, this must be the case for targets of KSHV miRNAs.

**Figure 9:**
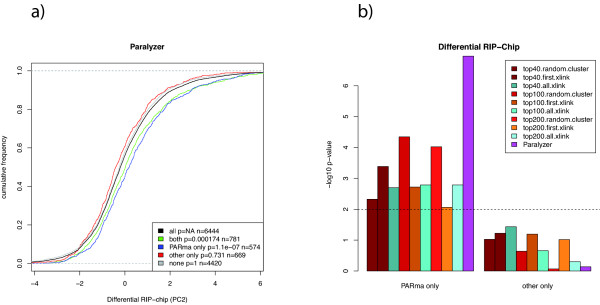
**Validation against RIP-Chip**. **(a) **The distribution of differential RIP-Chip enrichments (PC2 scores) of BCBLl and DG75 for different sets of PAR-CLIP targets. Higher values indicate a stronger enrichment of a gene with RISC in BCBLl than in DG75, and, therefore, a set of KSHV miRNA targets should have a right-shifted distribution of PC2 scores. Genes that have been identified by PARma as well as PARalyzer to be KSHV miRNA targets indeed show such a shift, as well as genes that have only been found by PARma and not by PARalyzer (PARma only). In contrast, genes that are not targets of KSHV miRNAs according to both PARma and PARalyzer do not show a shift. Interestingly, genes found exclusively by PARalyzer and not by PARma are not shifted as well. (b) The *P *values for the comparisons of PARma to all other methods indicate that PARma not only outperforms PARalyzer but all other methods as well.

Thus, we determined all genes that contain a KSHV miRNA target site according to PARma and PARalyzer (*both*), that contain a KSHV miRNA target site according to PARma and no KSHV miRNA target site according to PARalyzer (*PARma only*) and that contain a KSHV miRNA target site according to PARalyzer only (*PARalyzer only*) and compared it to genes without KSHV miRNA target sites (*none*); see Figure 9a. The *both *and *PARma only *genes showed significantly elevated differential RIP-Chip enrichment values (*P *< 2 × 10^−4 ^and *P *< 2 × 10^−7^, respectively, one-sided Kolmogorov-Smirnov test), whereas *PARalyzer only *and *none *genes were indistinguishable from the background. Thus, based on the RIP-Chip data, PARma effectively gets rid of false positive target sites detected by PARalyzer, and, in addition, picks up false negatives not detected by PARalyzer. We also repeated the same analysis for other methods replacing the PARalyzer results with similar results (see Figure 9b).

## Discussion

### PAR-CLIP clusters

In this paper, we present an in-depth investigation of seed sites in PAR-CLIP clusters. The standard approach to assign miRNAs in all PAR-CLIP studies published so far [23,25-27] is to select the top *N *expressed miRNAs and identify seed sites in the respective PAR-CLIP clusters. However, it is not clear, how *N *should be chosen: for small *N*, only a small fraction of clusters can be assigned and for larger *N*, cluster assignments are more and more ambiguous. Furthermore, independent of the choice of *N *or the exact way of searching for seeds, miRNA expression correlates only poorly with the number of clusters. Also, multiple studies report small RNAs other than miRNAs associated with the RISC. Thus, it seems advantageous not to restrict the search to a predefined set of miRNA seeds. PARma can be used for both searching for a predefined set of seeds and for an unconstrained search for all possible k-mers. In both cases, the assigned seeds fulfill two conditions in each cluster: first, the observed T to C conversions and RNase cleavage sites relative to the seed position match a model derived from all clusters and second, the seed site sequence is overrepresented. As illustrated in Figure 8, the unrestricted search is even more accurate in terms of assigning KSHV or EBV miRNAs to clusters that are exclusively present in KSHV or EBV infected cells, respectively.

We propose that the general approach of PARma can also be applied to other kinds of CLIP data. For instance, for iCLIP data [22], reads in valid target sites should start immediately after cross-linking sites. These specific start positions could be incorporated into an iCLIP model instead of the PAR-CLIP model of conversions and RNase T1 cleavage sites. However, how effective it is to exploit these characteristics of iCLIP data remains to be seen as more and more iCLIP data becomes available.

Clusters from a CLIP experiment are not necessarily true binding sites of the protein of interest: neither the immunoprecipitation (IP) step nor the gel separation are 100% specific and thus, there may be artifacts of other RNA binding proteins (RBPs). If 40 distinct miRNA seeds are considered and matched to such clusters, more than 20% of the unspecific clusters are expected to contain at least one seed match by chance (assuming an average cluster length of 30 bp and a seed length of six). This increases to almost 70%, when 200 miRNA seeds are considered. Thus, we expect that there are a considerable number of false positive miRNA target sites in current PAR-CLIP datasets. Finding a reliable way of scoring clusters in order to filter out such false positives is therefore of great importance.

To our knowledge, PARma is the first method to provide a scoring system that has been proven to improve accuracy upon filtering. The rationale for that is that there is no reason why unspecific clusters should match our PAR-CLIP model. Indeed, Cscores of intronic clusters, which likely are the result of unspecific IPs of other RBPs, are significantly lower than Cscores of 3' UTR clusters (data not shown) in both AGO-PAR-CLIP datasets, which is in agreement with known mechanisms of miRNAs. Furthermore, even if unspecific clusters match the PAR-CLIP model by chance and contain active k-mers by chance, it is unlikely that these k-mers occur at a position that matches the model. Thus, both Cscore and MAscore are expected to improve accuracy (see also Figure 8c and 8f).

### PARma

For the conversion model used in PARma, we assume that cross-linking events are independent of each other. This means that given a uridine at a certain position relative to the seed site, the probability that a cross-linking event takes place and is sequenced at this position is not dependent on the location of other uridines. This assumption may be wrong if one of the other uridines is already cross-linked. However, the probability that two cross-links occur in close vicinity to each other is very low, since the incorporation rate of 4-thiouridine (4sU) is only about 1/40 and only 4sU is cross-linked with high frequency at the wavelength used in PAR-CLIP [23]. In addition, the reverse transcriptase (RT) is known to be rather inefficient in reading through the peptide chain still cross-linked to the 4sU-residue (which is responsible for the U to C transition). Therefore, it becomes rather unlikely that the RT reads through two cross-links in a single RNA fragment.

Note also, that the model for conversions is not simply built by summing all cross-linking events for each position globally over all clusters. Such a procedure would be heavily influenced by a few clusters that have thousands of reads in comparison to the many clusters having only a few dozen reads. In contrast, our parameter estimation for the conversion model does not only exploit all clusters, but is also robust against outlier clusters by using robust regression and quadratic programming. Robustness in the parameter estimation is an important issue, especially in the initial iterations. This is because seeds are not yet assigned with high confidence leading to many outliers.

PARma does not necessarily assign seed sites directly downstream of cross-linking sites. When the next uridine upstream of a true seed site is several nucleotides away, it may still become cross-linked. In this case, PARma may still find another k-mer closer to the cross-linking site, dependent on the sequences, on other cross-linking events in the same cluster and on the RNase cleavage sites. However, PARma will report a low MAscore, since the other position will score similarly well.

PARma can be run for different values of *k*. The smallest reliable seed used in the literature is miRNA bases two to seven [2,9-12]. However, we noticed that PARma with *k *= 6 resulted in slightly worse accuracies for both our data sets in comparison to *k *= 7 (data not shown). This may be a consequence of the fact that random six-mers are expected to occur every 4,096 bases, and thus, approximately every 100 clusters (the median length of clusters is 47). When at least 100 miRNAs with different six-mer seeds are considered, every single cluster would on average have a seed match by chance. Thus, kmerExplain may have difficulty in reliably extracting the signal of overrepresented six-mers.

Because of the requirement that only a single k-mer can explain a cluster, kmerExplain is able to avoid overrepresented partial k-mers: consider the 7mer-A1 seed site UCGUCGA that explains hundreds of clusters. Obviously, the sequence CGUCGAG is expected to be present in 1/4 of these clusters and is thus highly overrepresented in the collection of all clusters. This overrepresented partial k-mer may also occur in additional clusters, that is, without the leading U. Even if it is not overrepresented by itself but only due to an overlapping k-mer that is truly overrepresented, all additional occurrences may be mistaken for the seed site of a targeting miRNA not because the miRNA is active but only because of the overlap to an active miRNA seed. Obviously, kmerExplain avoids such overrepresented partial k-mers because of the requirement that only a single k-mer can explain a cluster.

### Comparison with PARalyzer

PARalyzer is a software package specifically designed to analyze PAR-CLIP data [28]. It utilizes kernel density estimationg to compute probabilities of interactions along each cluster based on the normalized numbers of conversions and non-conversions at each position. There are two main differences to the basic approach used by Hafner *et al. *[23]: first, an interaction site is identified when the estimated density of conversions is greater than the estimated density of non-conversions instead of using the main cross-linking site for all clusters, which are filtered by specific criteria. Second, due to the kernel, the neighborhood of uridine sites is incorporated using an arbitrarily chosen bandwidth parameter. It is unclear whether this approach is able to filter out unspecific clusters effectively. In addition, the pattern discovery module does not incorporate information about cross-linking or RNase cleavage positions and is, thus, unable to resolve and score ambiguous seed matches. Furthermore, the PARalyzer pipeline does not include a method to handle spliced reads and, therefore, all studies that have used PARalyzer [25,27,28] may have missed all target sites that span exon-exon junctions. In the datasets we analyzed, 22.4% of all clusters in the coding region of transcripts span splice junctions (about 6% of all clusters).

### Differential PAR-CLIP

In order to evaluate PARma, we directly compared the number of PAR-CLIP sequencing reads from multiple experiments mapped to each individual cluster. Our evaluation is based on the following consideration: when a cluster is a valid target site of a KSHV miRNA, for instance, AGO should not be associated with it in KSHV negative cells and, therefore, the corresponding PAR-CLIP experiment should not yield sequencing reads mapping to this cluster and so it is an exclusive cluster.

While this is true for approximately 80% of all clusters assigned to a KSHV or EBV miRNA in both of the respective datasets, when PARma is used (see Figures 8b and 8e), there is a considerable number of clusters where this is not true. There may be several reasons: first, there is a considerable amount of background in the data, that is, sequencing reads not due to specific cross-linking to AGO. Indeed, almost all clusters have a positive log_2 _fold change of PAR-CLIP reads, which may be a consequence of background. Second, a target site could be targeted by multiple miRNAs. This is very probable for seed homologous viral miRNAs (for example, kshv-miR-K12-11 has the same seed as hsa-miR-155), but may also occur when there are strongly overlapping target sites. Accuracy increases when clusters are filtered by MAscore (see Figure 8c and 8f), which also indicates ambiguous assignments. Third, clusters may not be valid target sites and just by chance contain seeds of KSHV or EBV miRNAs, and, as a consequence, accuracy also increases when clusters are filtered by Cscore.

It would be of great benefit to be able to convert our scores to a false discovery rate as a statistically meaningful measure. This could be done if there was a way to determine how many of the non-exclusive clusters are still valid KSHV or EBV target sites. However, it is difficult to estimate the background, which is dependent on transcript expression, on other RNA binding proteins that target these transcripts and probably on many more factors. Additionally, the extent of overlapping or truly ambiguous target sites is unclear. Furthermore, the presence of reads is subject to stochastic sampling effects due to the relatively small numbers of reads. Thus, it is currently not possible to estimate reliable false discovery rates based on differential PAR-CLIP.

## Conclusion

In this paper we presented PARma, a method to analyze PAR-CLIP data. Clusters are defined in a similar way as before [23,28]. The main purposes of PARma are (a) to define reliable miRNA target sites and (b) to identify the miRNA responsible for each identified target site. Therefore, two scores are computed: the Cscore assesses the likelihood that a cluster is a valid miRNA target site and the MAscore corresponds to the confidence that the assigned miRNA is the true regulator.

PARma utilizes features specific to PAR-CLIP data to determine seed sites: the positions of cross-linking sites and missing cross-links as well as cleavage sites of RNase T1 relative to seed sites are learned and incorporated into a generative model. This model is used to guide a novel pattern discovery tool, kmerExplain, that estimates activity probabilities for k-mers.

Our method can be used to discover active k-mers in an unbiased manner, that is, without assuming a set of admissible k-mers such as the top *N *miRNA seeds. Each reported active k-mer nevertheless has two properties: it explains several clusters and the positions where it occurs match the model of PAR-CLIP data learned from all target sites. Using differential PAR-CLIP data, we have shown that PARma is more accurate than existing methods and that both Cscore and MAscore are useful measures to rank clusters.

## Methods

### Data

The data from Gottwein *et al. *[25] has been downloaded from GEO (accession number: GSE32113). DG75 and BCBL1 PAR-CLIP experiments have been performed as described [43,44]. Briefly, a total of 3 × 10^8 ^cells per replicate were grown and treated with 4-thiouridine (Sigma) for 14 hours (final concentration 100 μM). Cells were pelleted and washed in cold phosphate buffered saline (PBS). Aliquots of 5 × 10^7 ^cells were resuspended in 5 ml of cold PBS, placed in a 15 cm petri dish and irradiated at 365 nm with 100 mJ twice on ice, with a 30 s break in between. Cross-linked cells were collected, pelleted and snap-frozen. PAR-CLIP was performed using 11A9 anti-Ago2 monoclonal antibodies [45]. PAR-CLIP sequencing data have been deposited at GEO (accession number: GSE43909).

### Raw data processing and cluster definition

The deep-sequencing data were processed using an in-house pipeline consisting of adapter trimming, read mapping against genomes and transcriptomes, integrating all mappings and cluster identification as well as filtering.

#### Read mapping

The 3' sequencing adapter sequence were trimmed from each sequencing read using a specially tailored sequence alignment variant that aligns a prefix of the adapter sequence to a suffix of each sequencing read. After that, equal sequences are collapsed and mapped to the human genome (hg19), the KSHV genome (NC 009333.1), the EBV genome (NC 009334.1) and the human transcriptome (Ensembl v60) using Bowtie version 0.12.7 [46]. For each collapsed read, all mappings for an experiment were then collected and the best in terms of mismatches were written to a single BED file for each experiment including information about the read count (number of sequences before collapsing), the mismatches of each alignment and the number of alignments after mapping transcriptome alignments to the genome. Here, T to C conversions were not counted as mismatches, since they were expected due to the experimental protocol.

#### Cluster identification

All BED files were then simultaneously scanned chromosome by chromosome in a strand-specific manner and overlapping reads were clustered. We used only reads without mismatches (except for T to C conversions). Clusters were then filtered according to similar criteria as before [23,28]: read count at least five and at least three distinct read species. Clusters were quantified using the count of the main cross-linking site. After clustering, normalization factors were computed such that the median fold change to a reference experiment (we took the one with the most reads) was one. Then, in a second pass, all clusters were removed where all experiments had less than ten normalized read counts.

We also implemented three additional options: first, it is known that two target sites may overlap. Especially for viral miRNAs, several such cases are known [38]. Thus, we split each cluster: only reads spanning the main cross-linking site were used and the criteria from above were checked. Then, the main cross-linking site of the remaining reads was determined. This was repeated as long as all criteria were fulfilled. Second, since target sites may span splice junctions and we mapped reads to the transcriptome, we can also identify spliced PAR-CLIP clusters. However, when allowing for spliced reads, the definition of a cluster is not straightforward: for instance, for a 3' end of an exon, there may be reads starting in the exon and ending in the neighboring intron and reads that connect this exon to various other exons. We resolved such inconsistencies by first removing all exon-intron reads and then by removing reads to exons with fewer reads, if necessary.

Third, since target sites may be wider than the maximal sequence length, we extended all untrimmed reads up to the next RNase T1 cleavage site (that is, after the next G). This is important because in the following, we specifically use these cleavage sites in our generative model.

#### Visualization

In order to visualize PAR-CLIP data appropriately, we developed a specialized web-based visualization tool (see Figure 2). Unlike the widely used genome browsers from UCSC or Ensembl, our viewer has specialized visualization tools for PAR-CLIP data: we can visualize several evolutionary conservation scores, including k-mer branch lengths that have been used for miRNA target prediction [11], sequence read coverage, SNPs, the actual reads with indicated conversions, conversion densities, transcripts and PAR-CLIP clusters. Unlike genome browsers, our viewer is able to shrink introns in a data-dependent way (that is, if there are no reads mapped to an intron, it is not visualized at the same scale as the exons but shrunk to a few pixels). This is a major advantage over showing everything at the same scale when visualizing transcript-related data, since usually the long introns are often not of interest in contrast to the short exons.

### PARma

The result of our preprocessing, which is very similar to previous work [23,28], is a set of clusters  L. Each cluster L∈L is characterized by its sequence *s*(*L*), its conversion profile *conv_L _*and two vectors *start_L _*and endL. *conv_L _*is a vector containing for each position within *L *the number of conversions, whereas *start_L _*and *end_L _*contain for each position the number of reads starting and ending there, respectively. Furthermore, we define *T*(*L*) = {*i *∈ {1...|*s*(*L*)|} | *s*(*L*)*_i _= T*} as the set of possible conversion sites and *G*(*L*) = {*i *∈ {1...|*s*(*L*)|} | *s*(*L*)*_i _*= *G*} as the set of possible RNase T1 cleavage sites.

#### Model fitting

The PARma model consists of three submodels, incorporating T to C conversion data, 5' RNase cleavage data and 3' RNase cleavage data. The conversion model assigns a cross-linking probability *xlink*(*i*) to each position *i *relative to the seed site. Then, the cross-linking score *s_xlink _*for a seed position *j *in cluster *L *can be computed as:

$$s_{xlink}\left(L,j\right)=\frac{\underset{k\in T\left(L\right)}{\sum }conv_{L}\left(k\right)\cdot xlink\left(j-k\right)}{\underset{k\in T\left(L\right)}{\sum }conv_{L}\left(k\right)\cdot \underset{k\in T\left(L\right)}{\sum }xlink\left(j-k\right)}$$

This is essentially the normalized dot product of two vectors: the first vector contains the observed conversion counts for all conversion positions, the second contains the cross-linking probabilities for these positions. Thus, *s_xlink _*(*L*, *j*) = 1 if and only if the observed conversions exactly meet the expected cross-links and approaches 0 when the observed counts differ from the expected. Note that *xlink *can only be known up to a constant factor. This allows us to fit the model without making any further assumptions: given a current estimate *j *of the seed position for each cluster *L*, we first estimate the ratio *R_k,l _*for each pair of model positions *k *and *l *by collecting all clusters *L *with *j *− *k *∈ *T*(*L*) and *j *− *l *∈ *T*(*L*). Then we use robust linear regression to fit a line through the origin given the values *conv_L _*(*j *− *l*) and *conv_L _*(*j *− *k*) of all collected clusters *L*. The slope of this line then is a robust estimate of *R_k,l_*. Given the estimates of *R_k,l _*for all *k *<*l*, we obtain the final estimate of *xlink *by minimizing:

$$\underset{k,l}{\sum }{\left(\frac{xlink\left(k\right)}{xlink\left(l\right)}-R_{k,l}\right)}^{2}$$

subject to *xlink*(*j*) ≥ 0 and Σ*_j _xlink*(*j*) = 1 using quadratic programming. Note that the final constraint arbitrarily fixes the above mentioned constant factor and is necessary to get a unique solution.

The 3' RNase cleavage model assigns the cumulative probability *c*3(*i*) that the RNase cleavage site is ≤*i *to each position *i *relative to the seed site. Given a cluster *L*, let *G*(*L*) = {*k*_1_, ..., *k_n_*} with *k_i−1 _*<*k_i_*. Then, the downstream cleavage score *s*_downstream _for a seed position *j *in cluster *L *can be computed as:

$$\begin{array}{c} s_{\text{downstream}}\left(L,j\right)=\frac{\underset{i\in 1...n}{\sum }end_{L}\left(k_{i}\right)\cdot p\left(k_{i}\right)}{\underset{i\in 1...n}{\sum }end_{L}\left(k_{i}\right)}\\ p\left(k_{0}\right)=c3\left(j-k_{0}\right)\\ p\left(k_{i}\right)=c3\left(j-k_{i}\right)-c3\left(j-k_{i-1}\right) \end{array}$$

Note that we use cumulative probabilities here: in contrast to cross-linking positions, RNase cleavage sites are not independent. For instance, let cluster *L*_1 _have two consecutive G's 5 bp downstream of the true seed site (= *SEED = NNNNNGG*...) and cluster *L*_2 _only one G 6 bp downstream of its true seed site (= *SEED *= *NNNNNNG*...). The second G in *L*_1 _is at the same position relative to the seed site as the single G in *L*_2_. The RNase may have enough room to cut after the first G in *L*_1 _and thus all reads in *L*_1 _may end 5 bp downstream of the seed site. In cluster *L*_2_, all reads will end 6 bp downstream of the seed site. Thus, depending on where other G sites are located, read end probabilities will differ. Using cumulative probabilities in the model and computing the probabilities depending on G locations from cumulative probabilities is able to alleviate this problem. *c*3 is estimated by using the current estimates *j *of the seed position for each cluster *L*. The cumulative probability then is the number of times a position is upstream of the main RNase cleavage site divided by the number of clusters.

The 5' RNase cleavage model is formulated analogously to the 3' model. The final score for a position *j *in cluster *L_i _*then is calculated as the product of the three submodel scores:

$$p_{i,j}=s_{xlink}\left(L_{i},j\right)\cdot s_{\text{downstream}}\left(L_{i},j\right)\cdot s_{\text{upstream}}\left(L_{i},j\right)$$

#### KmerExplain

Given a set of sequences $S=\left\{S_{1},\ldots ,S_{n}\right\}$ and scores *p_i,j _*for each position *j *in cluster *L_i_*, kmerExplain estimates k-mer activity probabilities. This is done using an EM algorithm, which iteratively applies expectation (E) and maximization (M) steps to estimate the parameters of a probabilistic model under some hidden variables. We derive an EM algorithm for the following model: we assume that each sequence is generated by only a single k-mer. Then, the probability of generating a sequence *S *by a k-mer at its *j*th position is:

$$P\left(S|j\right)=\alpha_{S^{j}}\cdot \underset{c\neq j}{\prod }\left(1-\alpha_{S^{c}}\right)$$

Here, *α_x _*is the activity probability of k-mer *x *and *S^j ^*denotes the *j*th k-mer in *S*. The likelihood of  S then is:

$$P\left(S\right)=\overset{n}{\underset{i=1}{\prod }}P\left(S_{i}\right)=\overset{n}{\underset{i=1}{\prod }}\underset{j}{\sum }P\left(S|j\right)p_{i,j}$$

Thus, we have to estimate *α_x _*for all k-mers *x *under hidden parameters *j *(the active k-mer position in *S_i_*). In the E step we compute the values *q_i,j _*given the current estimates of *α_x _*as:

$$q_{i,j}=\frac{p_{i,j}P\left(S_{i}|j\right)}{\underset{c}{\sum }p_{i,c}P\left(S_{i}|c\right)}$$

The values *q_i,j _*represent current estimates of the probability *P*(*j*|*S_i_*). In the M step the estimator for *α_x _*then is computed as:

(1) $$\alpha_{x}=\frac{1}{n}\underset{i,j}{\sum }q_{i,j}\cdot \delta_{x=S_{i}^{j}}$$

where *δ_x = y _*is the Kronecker delta such that *δ_x = y _*= *1 *if *x = y*. and *δ_x = y _*= 0 otherwise.

Proof: The conditional expected value of the log likelihood and its partial derivative with respect to *α_x _*are:

(2) $$E=\underset{i,j}{\sum }q_{i,j}\text{log}P\left(S_{i}|j\right)$$

(3) $$=\underset{i,j}{\sum }q_{i,j}\text{log}\left(\alpha_{S_{i}^{j}}\cdot \underset{c\neq j}{\prod }\left(1-\alpha_{S_{i}^{c}}\right)\right)$$

(4) $$\frac{\delta E}{\delta \alpha_{x}}=\frac{1}{\alpha_{x}}Q_{x}-\frac{1}{1-\alpha_{x}}Q_{\bar{x}}$$

(5) $$Q_{x}=\underset{i,j}{\sum }q_{i,j}\cdot \delta_{x=S_{i}^{j}}$$

(6) $$Q_{\bar{x}}=\sum_{i,j}q_{i,j}\cdot \left(1-\delta_{x=S_{i}^{j}}\right)$$

Since $Q_{x}+Q_{\bar{x}}=n$, setting (4) to zero and solving for *α_x _*yields equation (1). □

#### Final assignment and integration

The output of the final iteration consists of scores *p_i,j _*for each position *j *in cluster *L_i _*as well as *q_i,j_*, which are estimates of the probability *P*(*j*|*S_i_*). The first is a quantity indicating how well the experimental data fit the model when any k-mer at position *j *has generated cluster *L_i_*. The latter incorporates the k-mer activity probability, that is, how well the experimental data fit the model when the given k-mer at position *j *has generated cluster *L_i_*. Furthermore, for each cluster *L_i _*we get the most probable k-mer generating this cluster at position *g_i _*= argmax*_j _*{*q_i,j_*}.

We use these quantities to compute confidence scores for each cluster (Cscore) and each k-mer assignment (MAscore):

(7) $$\text{Cscore}\left(i\right)=p_{i,g_{i}}$$

(8) $$\text{MAscore}\left(i\right)=\frac{q_{i,g_{i}}}{\underset{j}{\sum }q_{i,j}}$$

We integrate multiple experiments (either replicates of the same condition or multiple conditions) by first running PARma for each experiment individually and then taking the generating k-mer by computing a weighted sum over all *q_i,j _*from all experiments (weighted by the respective read count in the cluster) and taking the maximum. The Cscore then is the weighted sum of the $p_{i,g_{i}}$ values and the MAscore the maximal MAscore of all experiments at this position.

## Software availability

PARma is published under the GNU General Public License v3 and is available as supplementary material (see Additional file 1) and from the project website [50].

## Abbreviations

AGO: Argonaute; Cscore: cluster score; bp: base pair; EBV: Epstein-Barr virus; EM: expectation maximization; HITS-CLIP: high-throughput sequencing of RNA isolated by cross-linking immunoprecipitation; iCLIP: individual-nucleotide resolution cross-linking and immunoprecipitation; IP: immunoprecipitation; KSHV: Kaposi's sarcoma-associated herpesvirus; MAscore: miRNA assignment score; miRNA: microRNA; PAR-CLIP: photoactivatable-ribonucleoside-enhanced cross-linking and immunoprecipitation; PARma: PAR-CLIP miRNA assignment; PBS: phosphate buffered saline; RBP: RNA binding protein; RIP-Chip: RNA binding protein immunoprecipitation followed by chip analysis; RIP-seq: RNA binding protein immunoprecipitation followed by sequencing analysis; RISC: RNA-induced silencing complex; RT: reverse transcriptase; SNP: single nucleotide polymorphism; UTR: untranslated region.

## Conflict of interest statement

None declared.

## Authors' contributions

FE conceived and implemented the method, carried out evaluations and drafted the manuscript. LD designed the experiments, contributed ideas for the method and helped to draft the manuscript. LJ carried out the PAR-CLIP experiments. RZ supervised the project and helped to draft the manuscript. All authors read and approved the final manuscript.

## Supplementary Material

Additional file 1 **Zip file containing the runnable PARma pipeline including documentation and source code**. Click here for file
